# Conversion therapy with immunotherapy plus chemotherapy achieves a pathological complete response in stage IIIC NSCLC

**DOI:** 10.3389/fimmu.2023.1268153

**Published:** 2023-11-03

**Authors:** Yu Fu, Weichen Duan, Ran Xu, Jiajia Chen

**Affiliations:** ^1^ Department of Thoracic Surgery, Shengjing Hospital of China Medical University, Shenyang, China; ^2^ Department of Oncology, Shengjing Hospital of China Medical University, Shenyang, China

**Keywords:** non-small cell lung cancer, neoadjuvant therapy, immunotherapy, chemotherapy, histologic transformation

## Abstract

As stage IIIC non-small cell lung cancer (NSCLC) is not recommended for surgical resection, the survival and prognosis for stage IIIC NSCLC remain poor. More powerful and individualized therapies are urgently needed to improve the prognosis of stage IIIC NSCLC. Recently, immunotherapeutics have been increasingly considered in the neoadjuvant therapy of NSCLC. This study presents a patient with stage IIIC NSCLC achieving a pathological complete response (pCR) following conversion therapy with immunotherapy plus chemotherapy. This case also presents a histologic transformation from squamous cell carcinoma to adenocarcinoma after prolonged progression-free survival (PFS) following surgery. Collectively, this case suggests that conversion immunotherapy with chemotherapy and subsequent surgery can be considered and benefits a subset of unresectable stage IIIC NSCLC.

## Introduction

Stage III non-small cell lung cancer (NSCLC), accounting for approximately 30% of all newly diagnosed NSCLC cases, has a poor prognosis due to its high clinical heterogeneity and limited therapeutic approaches (1, 2). As it is challenging to achieve R0 resection, stage IIIC NSCLC is not recommended for surgery. However, the survival and prognosis for stage IIIC NSCLC remain poor using systematic therapy, with a median overall survival (OS) of 12.6 months and a 5-year survival rate of approximately 13% (3). Currently, concurrent chemoradiotherapy (CCRT) followed by durvalumab is the standard treatment for unresected stage III NSCLC (4). However, stage IIIC was evaluated as a prognostic factor associated with shorter progression-free survival (PFS) in the PACIFIC-R trial (5). Thus, more powerful and individualized therapies are urgently needed to improve the prognosis of stage IIIC NSCLC.

Surgery remains the most effective form of controlling local NSCLC when complete resection is feasible. Thus, neoadjuvant therapy, which allows unresectable NSCLC to be resected and increases the pathological complete response (pCR) rate, is frequently used for stage IIIA-B NSCLC (6). Achieving pCR *via* neoadjuvant treatment frequently improves prognosis (7). However, preoperative chemotherapy was reported to attain a low pCR rate of less than 10% (8–10). In contrast, neoadjuvant immunotherapy combined with chemotherapy elevated the pCR rate to 24%-63% in NSCLC (7, 9). Thus, immunochemotherapy provided a feasible option for the conversion setting in unresectable locally advanced NSCLC. However, few publications have focused on conversion therapy for stage IIIC NSCLC, which is the focus of this case report.

As immunotherapeutics are increasingly considered in the neoadjuvant therapy of NSCLC, we present a patient with stage IIIC NSCLC achieving pCR following conversion therapy with immunotherapy plus chemotherapy. Additionally, this case also presents a histologic transformation from squamous cell carcinoma to adenocarcinoma after prolonged PFS after surgery.

## Case presentation

A 45-year-old male patient came to our hospital in February 2021 due to 2 months of coughing. The patient had no particular medical or family history of cancer. The Eastern Cooperative Oncology Group (ECOG) performance score was 0 for this patient. On physical examination, there were no enlarged superficial lymph nodes (LNs) on his body.

**Figure 1:**
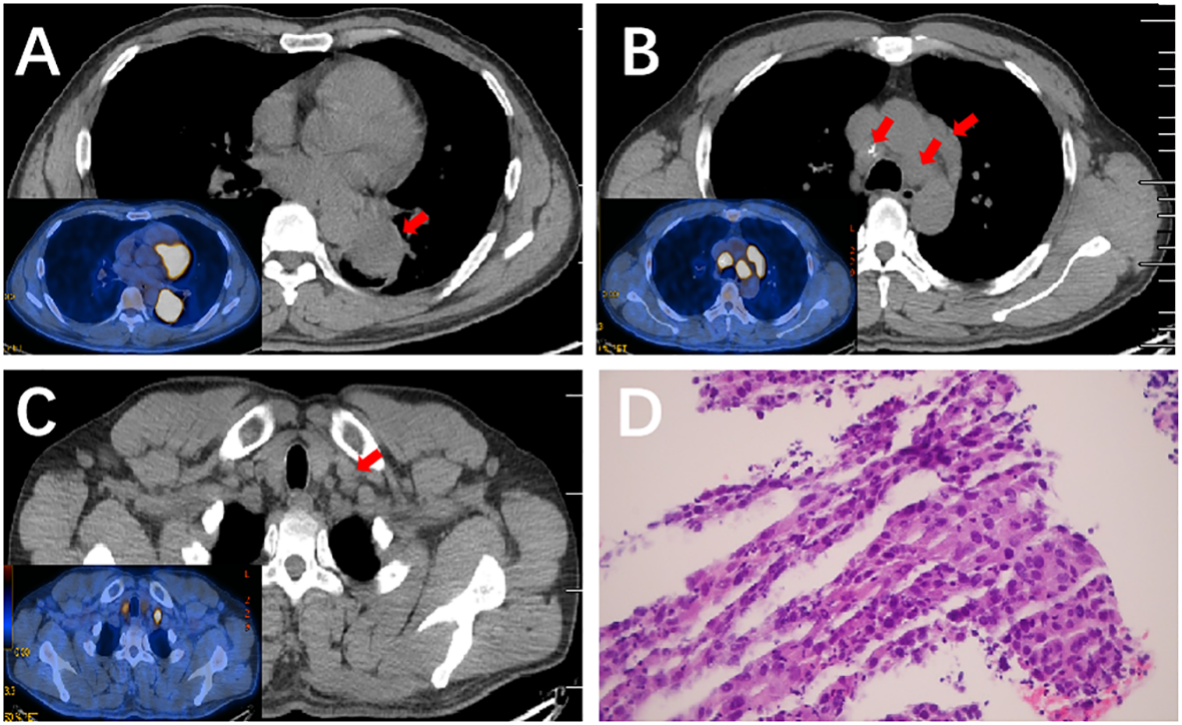
PET/CT scan of the lung and the pathological result of lung needle biopsy before therapy. **(A)** PET/CT scan of the lung showed a mass on the left lower lobe close to the left hilar with an increased uptake value (SUV=11.84). **(B, C)** PET/CT scan of the lung showed enlarged LNs with high metabolic activity in the mediastinal, and left supraclavicular regions. **(D)** Hematoxylin and eosin (H&E) staining (×200) of a lung needle biopsy showed poorly differentiated lung squamous cell carcinoma.

A positron emission tomography (PET)/CT scan (March 2021) found a 5.2 cm × 4.2 cm left lower lobe lesion close to the left hilar, with an increased uptake value (SUV=11.84) (**Figure 1A**). No invasion of the surrounding organ, pericardium, or pleura was observed. Additionally, enlarged LNs in the left hilar, ipsilateral and contralateral mediastinal, and left supraclavicular regions displayed high metabolic activity (**Figures 1B, C**). No distant metastases were observed. A lung needle biopsy showed poorly differentiated lung squamous cell carcinoma (**Figure 1D**). The patient refused further molecular pathologic analysis. Laboratory tests revealed levels of serum tumor markers: carcinoembryonic antigen (CEA) 0.66 ng/mL, cytokeratin-19 fragment (CYFRA 21-1) 5.7 ng/mL, serum neuron-specific enolase (NSE) 24.17 ng/mL and squamous cell carcinoma antigen (SCC) 3.8 ng/mL.

According to the 8^th^ American Joint Committee on Cancer (AJCC) classification (11), the patient was diagnosed with inoperable stage IIIC (cT3N3M0) lung squamous cell carcinoma. Currently, the standard treatment for inoperable stage IIIC patients is concurrent chemoradiotherapy followed by durvalumab (4). The patient was strongly willing to proceed with surgery. Whereas, surgical resection should not be performed and medical therapy should be considered for this patient according to the multidisciplinary team (MDT) discussion. Having discussed the potential risks of individualized treatment with the patient and his family, immunotherapy and chemotherapy were chosen. The patient was started with nanoparticle albumin-bound paclitaxel (nab-paclitaxel, 200 mg/m^2^) and lobaplatin (50 mg/m^2^) combined with tislelizumab (anti–PD-1 monoclonal antibody, 10 mg/kg), administered every 3 weeks for 2 cycles between March and May 2021. Immunochemotherapy was generally well tolerated without significant grade 3-4 adverse events except mild abnormalities in thyroid function.

**Figure 2:**
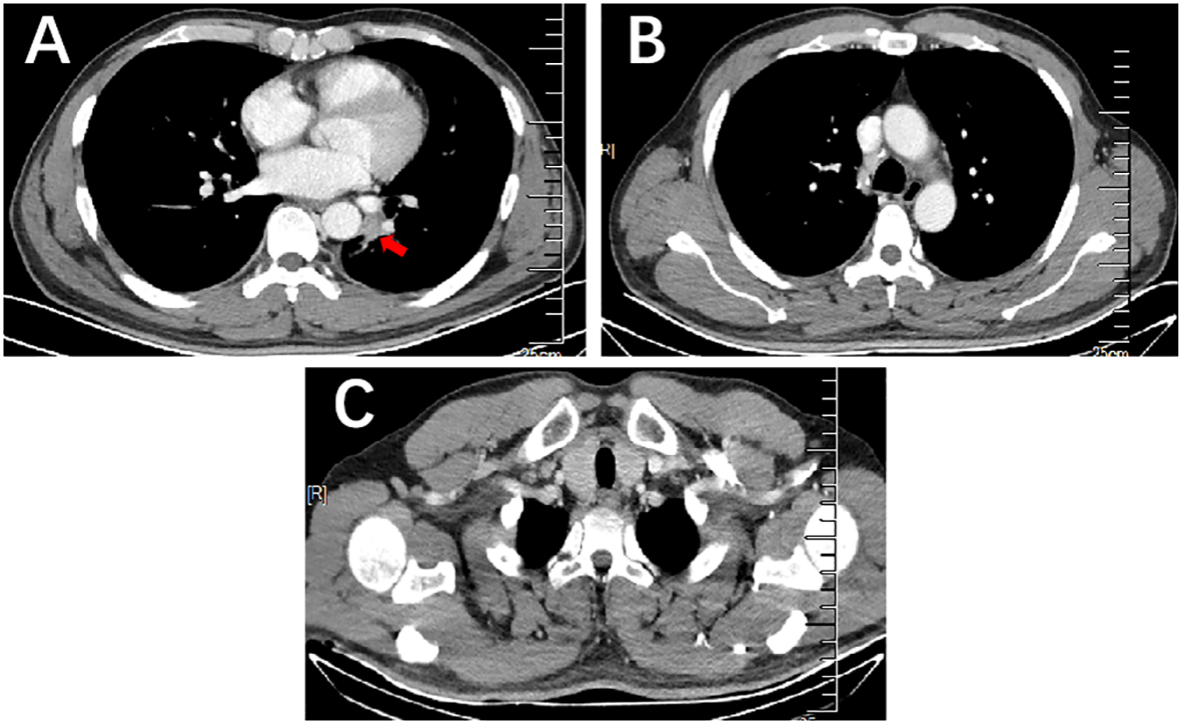
Enhanced CT of the lung mass with metastatic LNs after 2 cycles of immunochemotherapy. **(A)** Enhanced CT scan showed a decrease in mass size on the left lower lobe compared to that before therapy. **(B, C)** Enhanced CT scan showed previous metastatic LNs reduced to the standard size and presented normal morphology of reactive lymph nodes in the mediastinum and the left supraclavicular region.

**Figure 3:**
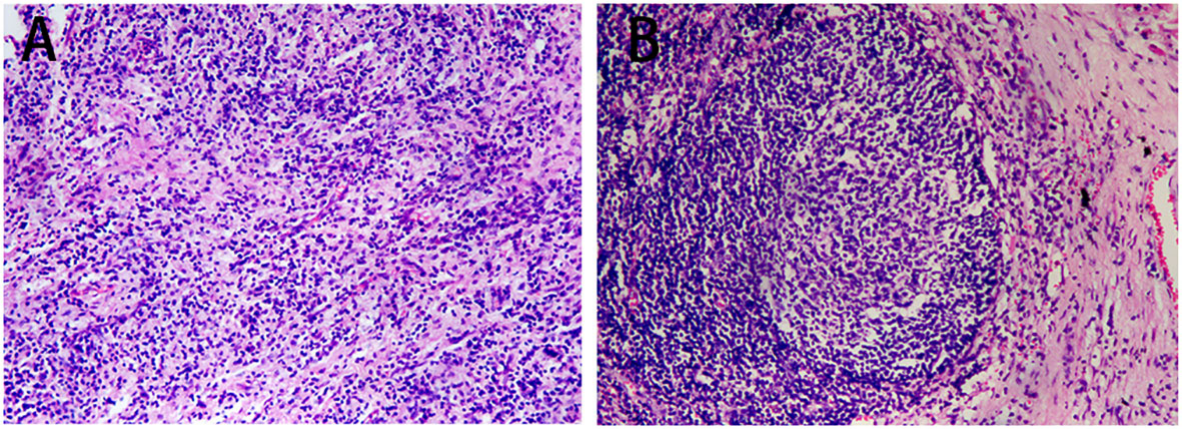
Postoperative pathology outcomes. **(A)** H&E staining (×100) of the tumor bed showed no viable tumor cells. **(B)** H&E staining (×100) of lymph nodes showed no viable tumor cells (station 4 left, 5, 6, 7, 9, 10, 11, and 12).

After 2 treatment cycles, the enhanced CT showed a decreased lesion (2.5 cm×1.4 cm) on the left lower lobe compared with the initial status (**Figure 2A**). Additionally, previous metastatic LNs were reduced to the standard size and presented normal morphology of reactive lymph nodes (**Figure 2B**). Besides, previously enlarged LN in the left supraclavicular regions was not detected (**Figure 2C**). Thus, the stage was improved to cT1N0M0. Based on the reported high pCR rate induced by neoadjuvant immunochemotherapy and the strong wishes for surgery of this patient, the MDT suggested thoracoscopic radical resection of the left lower lung tumor plus radical mediastinal lymph node dissection. Postoperative pathology showed no evidence of malignancy, and all lymph nodes (station 4 L, 5, 6, 7, 9, 10, 11, and 12) and margins were free of tumor cells (**Figures 3A, B**). The patient was determined to achieve pCR (ypT0N0M0) when assessed by pathologists. Considering the initial clinical stage of this patient, the MDT suggested 2 cycles of adjuvant immunochemotherapy (nab-paclitaxel d1+lobaplatin d1 + tislelizumab d1 q3W) and then administered with tislelizumab for up to 12 months. The patient only reported mild abnormalities in thyroid hormone levels without any other toxic side effects.

**Figure 4:**
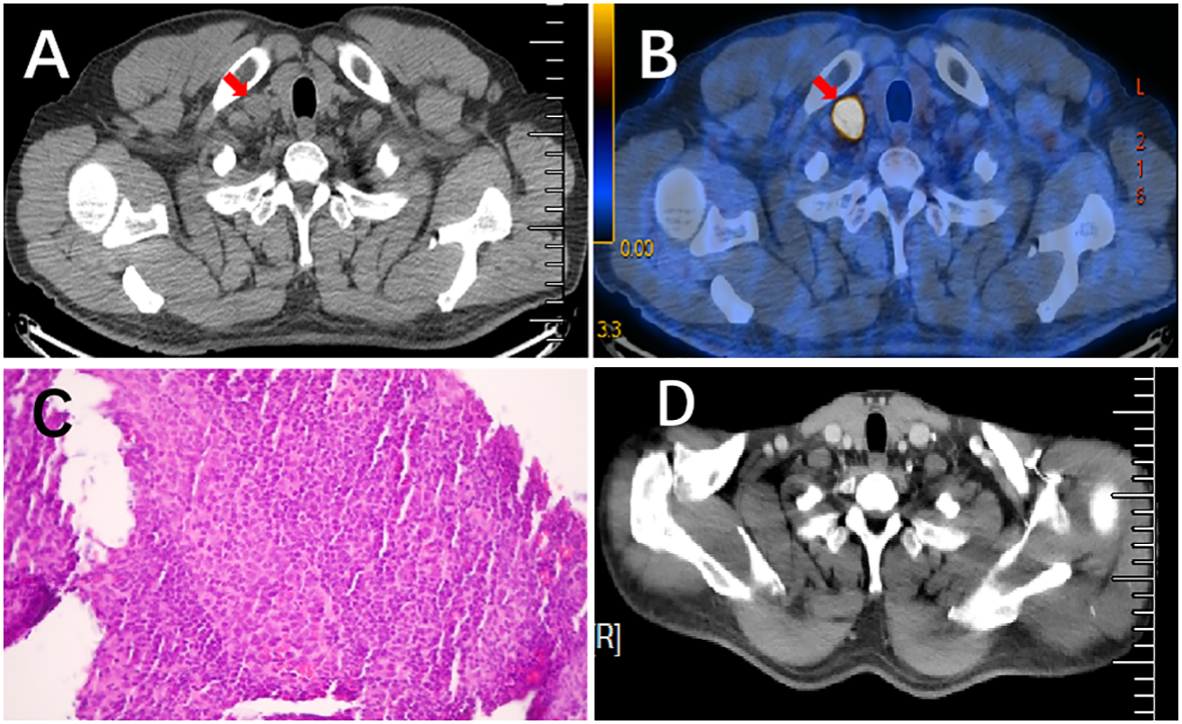
Pathological results of lymph node needle biopsy and the comparison of enlarged LN before and after treatment. **(A, B)** PET/CT scan showed an enlarged lymph node with high metabolic activity (SUV=21.57) in the right supraclavicular regions. **(C)** H&E staining (×200) of a lymph node needle biopsy showed metastatic, poorly differentiated adenocarcinoma. **(D)** Enhanced CT scan revealed that previously enlarged lymph nodes had disappeared after chemotherapy.

He was followed up for 20 months after surgery with no recurrence. In February 2023, subsequent PET/CT revealed an enlarged LN (approximately 1.7 cm×1.0 cm) with high metabolic activity (SUV=21.57) in the right supraclavicular zone (**Figures 4A, B**). Additionally, a pathologic example of this lymph node showed metastatic poorly differentiated adenocarcinoma (**Figure 4C**). The patient refused molecular analysis of the new biopsy specimen. Then, he received 4 cycles of chemotherapy with nab-paclitaxel and carboplatin. Enhanced CT in May 2023 showed the disappearance of the right supraclavicular LN (**Figure 4D**). Furthermore, the MDT suggested prophylactic radiotherapy (RT) to the mediastinum and bilateral supraclavicular zone to strengthen local control. The patient was under regular follow-up after completion of RT, and the PFS2 since then has not been reached.

## Discussion

Patients with resectable NSCLC who undergo R0 resection (lobectomy plus regional lymph node dissection) and achieve pCR have an increased likelihood of survival (7, 12). Recently, radical resectability was improved in locally advanced NSCLC due to superior downstaging *via* immunotherapy (7). Thus, chemotherapy combined with immunotherapy may provide an attractive opportunity for conversion therapy for stage IIIC NSCLC. However, there is still little data on processing surgery for previously unresectable stage IIIC NSCLC patients. In the current case, an inoperable NSCLC patient with stage IIIC successfully transformed to radical lung resection after conversion immunochemotherapy. The patient achieved pCR and had a favorable PFS1 of 20 months after surgery and has not reached PFS2 since the completion of chemotherapy and radiotherapy after relapse. It is encouraging that this conversion strategy may benefit a subset of stage IIIC NSCLC patients and enable them to undergo radical surgery.

Increasing evidence has demonstrated the feasibility and efficacy of neoadjuvant immunotherapy in locally advanced NSCLC. However, monotherapy with immune checkpoint inhibitors (ICIs) obtained a poor pCR rate of no more than 10% (13, 14). As the combination of immunotherapy with chemotherapy achieved additive or synergistic clinical activity (15), it has renewed interest in neoadjuvant immunochemotherapy in NSCLC. Published clinical trials evaluating neoadjuvant ICIs with chemotherapy significantly improved the pCR rate to 17.2%-63% (**Table 1**). Furthermore, the R0 resection rate of these studies was 83%-100%, representing a high complete surgical cure (**Table 1**). As higher pCR rate was associated with improved survival, neoadjuvant immunochemotherapy brings a better prognosis for stage III NSCLC. CheckMate 816, a phase 3 trial, showed longer median event-free survival in a nivolumab-based neoadjuvant regimen compared with chemotherapy alone (31.6 months *vs*. 20.8 months) in resectable IB-IIIA NSCLC (9). However, few random clinical trials have investigated the prognosis of conversion therapy for unresectable stage III NSCLC. Deng et al. retrospectively evaluated the feasibility of conversion immunochemotherapy in unresectable stage IIIB-IIIC NSCLC (n=51). The patients who underwent surgery had more favorable PFS than those in the non-surgery group (27.5 months *vs*. 16.7 months) (24). Thus, Deng’s study provides evidence for the feasibility and benefit of conversion immunochemotherapy in inoperable NSCLC. Similarly, two rounds of a PD-1 inhibitor with a chemotherapy regimen resulted in successful downstaging and achieved pCR in the current case. Encouragingly, the patient had a favorable PFS of 20 months after surgery, even greater than the median PFS (16.8 months) of unresectable stage III NSCLC patients in the PACIFIC trial (4). In addition to neoadjuvant immunochemotherapy, the synergistic effect of ICIs with other treatments in neoadjuvant settings, such as radiotherapy, anti-angiogenic therapy, interleukin-1β blockers, TIGIT inhibitors, multi-kinase inhibitors *et*c., has been evaluated in ongoing studies of NSCLC, which are summarized in **Table 2**. It seems that conversion therapy with ICIs plus other treatments might be a promising therapy for unresectable stage IIIC NSCLC.

**Table 1:** Summary of clinical evidence for neoadjuvant immunotherapy combined with chemotherapy in NSCLC.

Study name	Phase	Sample (n)	Stage	Neoadjuvant regimen	MPR %	pCR %	R0 resection%	Refs
CheckMate 816	3	505	IB-IIIA	Nivolumab+CT	36.9	24	83.2	(9)
NADIM	2	46	IIIA	Nivolumab+CT	83	63	89	(7)
AEGEAN	3	802	IIA-IIIB	Durvalumab+CT	33.3	17.2	94.7	(16)
Neotorch	3	404	II-III	Toripalimab+CT	48.5	24.8	95.8	(10)
KEYNOTE-671	3	797	II-IIIB	Pembrolizumab+CT	30.2	18.1	92	(17)
LungMark	2	100	II-IV	Tislelizumab+CT	74.4	48.7	97.4	(18)
NeoTPD01	2	33	IIIA-IIIB	Toripalimab+CT	66.7	50	96.7	(19)
TACT	2	35	IIIA-IIIB	Tislelizumab+CT	71.9	34.4	100	(20)
Shu et al	2	30	IB-IIIA	Atezolizumab+CT	57	33	87	(21)
Wang et al	2	33	IIIA-IIIB	Tislelizumab+CT	22.2	33.3	94.4	(22)
neoSCORE	2	60	IB-IIIA	Sintilimab+CT	41.4/26.9*	24.1/19.2^#^	91.7	(23)

CT, chemotherapy; MPR, major pathologic response; pCR, pathological complete response.

* MPR rate achieved 41.4% and 26.9% in 3-cycle neoadjuvant therapy group and 2-cycle neoadjuvant therapy group, respectively. # PCR rate achieved 24.1% and 19.2% in 3-cycle neoadjuvant therapy group and 2-cycle neoadjuvant therapy group, respectively.

**Table 2:** Ongoing clinical trials that included neoadjuvant combination therapy based on PD-1/PD-L1 inhibitors in NSCLC.

PD-1/PD-L1 Inhibitors	Medicines	Combination regimen of Neoadjuvant therapy	NCT Number/study name
**PD-1** **inhibitors**	Nivolumab	Com with Ipilimumab plus RT	NCT04933903
		Com with CT	NCT04025879/CheckMate 77T
	Sintilimab	Com with CT	NCT05244213, NCT04728724, NCT04326153, NCT04840290
		Com with CT plus Bevacizumab	NCT04973293, NCT03872661
	Toripalimab	Com with CT	NCT05800340
		Com with RT	NCT05798845
	Camrelizumab	Com with CT	NCT04943029, NCT04541251
		Com with Apatinib	NCT04506242, NCT04379739
	Pembrolizumab	Com with Lenvatinib	NCT04875585
		Com with CT	NCT04638582, NCT05894889
		Com with Ramucirumab	NCT04040361
		Com with Canakinumab	NCT03968419
	Tislelizumab	Com with CT	NCT05527808, NCT04379635/RATIONALE-315
		Com with Ociperlimab/LBL-007	NCT05577702
	Serplulimab	Com with CT	NCT05882513, NCT05775796, NCT05766800
	Penpulimab	Com with Anlotinib	NCT04846634
**PD-L1 inhibitors**	Durvalumab	Com with RT	NCT04245514
		Com with CRT	NCT05157542, NCT03871153 NCT04465968
		Com with Abequolixron plus CT	NCT05911308
		Com with Sirolimus	NCT04348292
		Com with Anlotinib plus CT	NCT04762030
	Atezolizumab	Com with CT plus Bevacizumab	NCT04512430
		Com with tiragolumab	NCT04832854
		Com with CRT	NCT04989283
		Com with CT	NCT03456063/IMpower030, NCT04865250
	envafolimab	Com with recombinant human endostatin plus CT	NCT05360979
	Sugemalimab	Com with CT	NCT05940532

Com, Combination; CT, chemotherapy; RT, radiotherapy; CRT, concurrent chemoradiotherapy.

After CCRT, in-field recurrence is a survival-associated negative prognostic factor for unresectable stage III NSCLC (25). However, an exploratory study for PACIFIC research revealed that most recurrent patients (approximately 80%) had their first progression inside the thorax (26). Kishi et al. also reported that in-field recurrence is the most common pattern of local recurrence in stage III NSCLC patients with CCRT/CCRT plus consolidative immunotherapy (27). Radiotherapy is effective for treating local relapse. However, the in-field recurrence pattern increases the complexity of reirradiation, which might provide only palliative benefit rather than meaningful survival prolongation (28). Thus, further strengthening of loco-regional control is still a major issue in the PACIFIC treatment model. In contrast, radical resection provides more powerful local control and determines long-term survival. Consistently, the patient had tumor progression beyond the original disease region without recurrence inside the thorax and mediastinum in the current case. The metastatic LN disappeared in this patient with sequential chemotherapy and radiotherapy, and the patient has had an unreached PFS2 since then. Collectively, our case suggests that conversion immunochemotherapy with pCR achievement might improve local-region control. For such non-in-field recurrence patterns, oncologists have more chances to accomplish local management, such as surgery and irradiation.

Despite our study indicating that conversion immunochemotherapy is practicable and safe for facilitating surgical resection in stage IIIC patients, this strategy may not benefit all cases due to high tumor heterogeneity. The response to ICIs determines the efficacy of conversion therapy. Thus, poor responders, such as non-smokers with EGFR mutations or ALK rearrangements, might be excluded (29). Recently, in addition to the PD-L1 level and tumor mutational burden (TMB), several novel molecular mutations, such as KRAS/TP53 mutations, BRAF mutations, LKB1/STK11 mutations *etc*., presented predictive value (30–32). However, most of these mutations are still under investigation, with a long way to clinical translational application. As the resection criteria are controversial among locally advanced lung cancer in the era of immunotherapy, the optimal assessment criteria for operability following conversion therapy are also urgently needed. Meanwhile, comprehensive evaluation and full MDT discussion are indispensable. Importantly, individualized conversion therapy can only be performed after complete communication with patients and obtaining patients’ consent.

Histologic transformation is a major mechanism of drug resistance in lung cancer patients. Small-cell lung cancer (SCLC) transformation has been reported frequently in NSCLC patients after targeted therapy (33). However, histologic transformation between adenocarcinoma and squamous cell carcinoma after ICIs treatment is unusual. In our case, histologic evolution from lung squamous cell carcinoma to adenocarcinoma was observed 6 months after the last tislelizumab treatment. The possible mechanisms for histologic transformation involve pluripotent cancer stem cell differentiation, tumor heterogeneity, selection of subclones that are unresponsive to ICIs over time, drug-induced immune escape mechanisms, and microenvironment interactions (34–37). However, the definitive mechanism is not clarified. Both ICIs and previous chemotherapy might be the reasons for adenocarcinoma transformation. Moreover, the limited specimens from the initial biopsy made it difficult to exclude a mixed tumor type at the first diagnosis. Thus, repeat biopsies can provide valuable information and should be encouraged to explore the mechanism of histologic transformation. However, more research is needed to clarify the underlying mechanisms of histologic transformation.

## Conclusions

We present a patient with initial unresectable stage IIIC NSCLC who underwent complete R0 resection and achieved pCR following conversion therapy with a PD-1 inhibitor plus chemotherapy. This case suggests that conversion immunotherapy with chemotherapy and subsequent surgery can be considered for a subset of unresectable stage IIIC NSCLC.

## Data availability statement

The original contributions presented in the study are included in the article. Further inquiries can be directed to the corresponding authors.

## Ethics statement

Written informed consent was obtained from the individual(s) for the publication of any potentially identifiable images or data included in this article.

## Author contributions

YF: Data curation, Formal Analysis, Writing – original draft. WD: Data curation, Formal Analysis, Writing – original draft. RX: Conceptualization, Data curation, Formal Analysis, Funding acquisition, Methodology, Project administration, Resources, Supervision, Writing – original draft, Writing – review & editing. JC: Conceptualization, Data curation, Formal Analysis, Funding acquisition, Methodology, Project administration, Resources, Supervision, Writing – original draft, Writing – review & editing.
